# Ghostwriters of crime narratives: Constructing the story by referring to intercept interpreters’ contributions in criminal case files

**DOI:** 10.1177/17416590221133304

**Published:** 2022-11-02

**Authors:** Nadja Capus, Cristina Grisot

**Affiliations:** University of Neuchâtel, Switzerland; University of Zurich, Switzerland

## Introduction

In the French film *La Daronne* (2020), directed by Jean-Paul Salomé, the impressive actress Isabelle Huppert plays an intercept interpreter: she works for the drug squad in Paris and interprets the secretly surveilled phone calls of drug dealers from Arabic to French. It is an exhausting job, and she is overworked and underpaid. To make matters worse, she is under pressure to pay for her mother’s expensive nursing home. One day, she becomes involved in a failed drug deal and has access to many kilograms of cannabis. While she continues her job with the drug squad, she changes sides and becomes a drug dealer herself. It is only when the police involved another interpreter in the surveillance process that her disloyalty is revealed. The movie foregrounds how dependent the police are on the intercept interpreter and her important, active role in making a criminal case. In fact, through her interpretation, Huppert does not merely mechanically translate one Arabic word into a French word. As an intercept interpreter, she does what intercept interpreters do: they select information, decipher codes, attribute importance to some parts of the intercepted conversation, neglect other parts, paraphrase or summarize sequences of the intercepted conversations, add contextual knowledge, provide their own interpretations of unclear statements when transforming them into written transcripts of translated wiretap records (TWRs; Capus and Havelka, 2022; Drugan, 2020: 314; González Rodríguez, 2015: 114; Nunn, 2010; Park and Bucholtz, 2009: 31). Intercept interpreters often (but not exclusively) present the content of a TWR as a seemingly word-for-word translation and transcription of original conversation that is structured as a dialog between two participants.

Moreover, they guide the drug squad, indicating when a handover will take place and when the police should move out for an arrest, a house raid, or to seize drugs. In the movie, Huppert gives hints (sometimes misleading ones) and shapes the course of the investigation in a dynamic exchange with the police. Obviously, prosecuting harmful behaviors such as those considered to be drug trafficking, terrorist activities, human trafficking, and organized crime by using legal means of intercepting communications would simply not be possible without intercept interpreters providing special language and situational knowledge. Intercept interpreters are the only ones who understand the content of conversations that potentially incriminate people. In drug-trafficking cases, these interceptions are also important to identify the organization and severity of the trafficking in terms of quantity and time span and to determine the role of those involved (Calderoni, 2012; Campana and Varese, 2012). What foreign-language speakers do is more than render a mere mechanical translation. In fact, they must interpret conversations and these interpretations provide the grounds for both, the prosecutor and the defense, to construct the prosecutorial master narrative and to develop the defense’s counter-narrative. Hence, there is no storytelling without the intercept interpreter.

In our paper, we examine the role of intercept interpreters in this storytelling. To do so, we employ the framework of narrative criminology, which has demonstrated the importance of stories for understanding both crimes and justice (Fleetwood and Sandberg, 2022). Narrative criminologists use the concept of narrative to analyze criminal behavior and processes of de/criminalization (Presser, 2009: 178). Research stemming from this theoretical framework focuses on offenders’ storytelling (Brookman, 2015; Fleetwood, 2016; Ioannou et al., 2017; Presser, 2009; Sandberg, 2013; Sandberg and Fleetwood, 2017), as well as that of victims (Cook and Walklate, 2019; Hourigan, 2019), criminal justice agents such as prosecutors (Offit, 2017, 2019), police or probation officers (Guldborg, 2016; Kurtz and Upton, 2017, 2018; van Hulst, 2013, 2014), and trial actors (Houge, 2019). It reveals that narratives impact both the proceedings of actions and our understanding of these actions. This approach is fruitful when it comes to studying the role of intercept interpreters in criminal proceedings because it offers a good opportunity to examine the narrative that the criminal justice agents officially responsible of enforcing criminal law (i.e. the police, prosecutors, and courts)—the so-called “storytelling animals” (Barrera, 2019)—are keen to tell about the prosecuting drug trafficking or other crimes. This narrative adds to the constitution of the meaning of the actions and activities that intercept interpreters perform as actors in the legal process. Hence, once the narrative is revealed, one question arises: what is the effect of telling a story in this way? In our view, the answer is that the way in which stories about investigating, prosecuting, and judging are told can influence in several ways the understanding of the intercept interpreters’ role in a criminal procedure. First, it could restrict their role to invisible ghostwriters, which would confirm the fictional idea of intercept interpreters as pure transmitters of language. Second, it could lead to ignoring or to misunderstanding their role. Third, it could break down the barriers of traditional approaches by making their contribution visible and transparently demonstrate the unique role they play while intercepting and interpreting.

Indeed, in the traditional body of research about interpreters in general, it is assumed that the role of intercept interpreters is nothing more than that of a mechanical, neutral, and accurate language converter (Angelelli, 2004: 2), a “meaning grasper or language modem” (Angelelli, 2004: 92: 92). The narrative that criminal justice agents deploy might blur or downplay the active role of intercept interpreters by presenting them as conduits or ghosts (idem: 14, 20; Nakane, 2009). More recently, our previous research has shown that the intercept interpreter’s mission and responsibility regarding the selection and editing of relevant information is only implicit, and case law obscures both this implicit mission and the investigators’ dependence on the interpreter’s translational activity (Capus and Griebel, 2021). Although intercept interpreters substantially help criminal justice agents in constructing a criminal case, thereby shaping their understanding of the events and actively contributing to the employment, criminal justice agents still adhere to the fictional idea that intercept interpreters solely produce literal translations. In other words, they perform only at the superficial level of the text without any obvious deeper intervention (Capus and Griebel, 2021: 92). As we noted above, however, intercept interpreters perform many other tasks. In doing so, they exercise agency and thus gain authorship status (Angelelli, 2004: 76). Their agency might, however, be both constrained by their positioning as “embedded” in the investigation and concealed by various textual strategies used in different types of documents, such as police reports or indictments.

Whereas previous research in the area of criminal justice focused on court rooms and police stations to study the making of the narrative, in this study we investigate the paperwork of case files. We then empirically analyze the mentioned textual strategies of criminal justice agents when referring to the contributions of intercept interpreters in order to examine whether the narrative deployed in the case file blurs or downplays the active role of intercept interpreters. Our study is based on material that derives from four case files from Swiss prosecutors in two French-speaking cantons in Switzerland to which we were given complete access.^1^ This material and our narrative criminological approach will increase our knowledge about how to critically reconstruct the narrative deployed in the case file of a criminal procedure.

## Case files as a site of storytelling

In the above-mentioned movie, the work of criminal justice agents with intercept interpreters was well informed and fact based. Nevertheless, there is one aspect that the movie does not show: paperwork. In fact, in practice, intercept interpreters do not appear before courts or orally report on what they hear while surveilling and interpreting intercepted conversations in trials. Hence, intercept interpreters are quite invisible in criminal proceedings. They are only partially and indirectly rendered visible by means of the documents they establish, the TWRs, or whenever crime file-makers, such as police officers, prosecutors and judges, explicitly refer to them in their reports, indictments, and judgments. This is the reason why the role of intercept interpreters may be studied only in the context of case files.

The perception of case files has undergone major changes in the past. In classical criminology, the prevailing idea was that files were a true reflection of reality (see Hermann, 1987: 44). Later, sociological work on record-keeping in institutional contexts (Garfinkel, 1967/1974; Smith, 1990; Zimmermann, 1974) has shown that case files are not objective descriptions of incidents, but rather serve the institution’s purposes. Hence, case files cannot be regarded as neutral representations of underlying phenomena: they are independent achievements of their authors. In other words, file documents are “active texts” (Smith, 1990) geared toward the needs of the criminal justice system. Instead of being a neutral conveyor of information, paperwork is a persuasive medium (Gibson et al., 2018). Documents are methodically designed to tell a story to an invisible and not-yet-present audience who will address the case later in the proceedings (Coulthard, 1996; Wolff, 2008).

We emphasize that in criminal procedures, the oral narrative is already a multimodal endeavor: on the one hand, speech acts are transformed into texts and vice versa, and on the other hand, speech acts themselves refer to or are based on previous paperwork (Scheffer et al., 2010: 18). Beyond that, however, it is also significant that some criminal proceedings essentially take place in writing. In fact, if no jury is used for criminal trials, trials in common-law jurisdictions largely depend on previously established paperwork, that is, the documentation made and included by police officials, prosecutors, and lawyers in the case file (Scheffer, 2006, 2007, 2010; Scheffer et al., 2010; Suresh, 2019). This paperwork approach is even more important in non-adversarial, inquisitorial civil law systems: the prosecutor—with the help of the police—is held to conduct an extensive pre-trial investigation to seek both incriminating and exculpatory evidence. This results in a first narrative written in the case file, which then will be handed over to the court (Grunewald, 2013: 381). Based on this case file, judges have the competence to render the final judgment or, alternatively, to decide that further investigation is needed. Hence, it is by no means an exaggeration to say that, in this context, the case file is at the heart of criminal proceedings (van Oorschot and Schinkel, 2015) and constitutes the blueprint of the narrative deployed during the criminal procedure.

In a case file, criminal justice agents collect heterogeneous documents pertaining to a criminal procedure. Hence, case files combine various acts authored by the criminal justice agents and are essentially dedicated to providing one powerful master narrative (Presser and Sandberg, 2015; Sandberg and Ugelvik, 2016). This narrative then legitimizes future actions and decisions, such as house-searching, seizures, arrests, undercover investigations, the final judgment, the sentencing, and the execution of such decisions. Often, criminal justice agents consider themselves not as telling a story “but rather as speaking in the universal voice” (Bandes, 1996: 385). Even though case files include documents (e.g. letters, interrogation records) that emphasize fragmented pieces of counter-narratives from defense lawyers, defendants, witnesses and others, new elements are treated in relation to the master narrative: they are oriented, examined, made plausible, recognized or rejected (Hanken-Illies, 2012: 295). During the whole procedure, the file is updated, and the narrative is developed step by step. This “unlimited capacity for addition and circulation transforms case files into a medium of presence” (Vismann, 2008: 10). The case file makes (presumably established) facts, activities and statements permanently available, and thus it integrates the temporally and spatially stretched procedure (Scheffer, 2008, 2012). In fact, it is the file that travels from one phase to the other of a procedure, from the police to the prosecutor and then to the court, and from one court to the next. In doing so, the case file allows the recycling of once-established facts at every stage of the legal process, from the very first police perception until the final judgment (Aronsson, 1991: 238; Capus, 2018: 206). In this way, a case file can tell “a story that transcends its body” (Kozin, 2007).

Nevertheless, storytelling through case files in criminal proceedings is a sophisticated task. Indeed, stories have more than one teller and are collaboratively and interactively constructed (see also Dollinger and Heppchen, 2019). This is an important aspect for our research about the contribution of intercept interpreters to the master narrative deployed in a criminal procedure. In fact, a criminal case files may combine: (i) police reports, (ii) written records of investigative interrogations with suspects or testimonies (Capus, 2018; Capus et al., 2014; Rock, 2017), (iii) reports by experts such as psychiatrists (Verde et al., 2006), (iv) paperwork accompanying procedural acts, such as application forms from prosecutors demanding judicial approval of certain coercive measures (e.g. wiretapping and detention), and (v) documents proving the execution of other coercive procedural acts (e.g. search and seizure). In the cases we showcase in this study, the case files also contain the TWRs that interpreters generated on the basis of selected parts of the monitored conversations. Within the case files, documents thus create “transdocumentary” knowledge, that is knowledge stemming from a visible or invisible relationship between texts (Bereswill et al., 2020: 206).

The narrative deployed by criminal justice agents through this paperwork can be characterized as having a dual-nature construction: while crafting the master narrative of suspected crimes, case file makers (police agents, prosecutors, and judges) simultaneously construct the narrative about the investigation, the prosecution and the judgment of criminal behavior. This dual nature recalls the duplicity of the narrative construction that Offit (2019) discovered when analyzing prosecutors’ development of their oral opening and closing statements before a jury. Prosecutors (and, in our study, also police officers and judges) are attentive to how their narratives might inform audiences’ perceptions of their own professional character and performance (Offit, 2019: 50 ss.).

## Method and material of the empirical study

Against this background, we quantitatively and qualitatively examine how case file makers tell stories by referring to the input of intercept interpreters as it is presented in the TWRs. To investigate this, we conducted one empirical study in which we used case files as databases. The cases stemmed from four closed criminal procedures about drug trafficking from two Swiss French-speaking cantons named here as A and B for anonymity reasons. They are part of a larger research project on the role of intercept interpreters in Swiss criminal proceedings, consisting of a corpus of 22 case files in total. It is important to note that it is the Swiss prosecutorial authorities who designated which case files can be included in our corpus. From this corpus, we selected the case files from the two French-speaking cantons that included the highest number of TWRs, two from each canton. In each case file, the police had intercepted the conversations of the suspected drug traffickers. The vast majority of the conversations were in a foreign language. The intercept interpreters then translated them into French and wrote them down, also in French, in the form of a TWR. Each of the four case file was examined and manually coded in its entirety; this resulted in a total of 9378 pages. The two case files from the canton A consist of 6125 pages, while the two case files from canton B consist of 3253 pages.

Table 1 provides an overall description of the four case files investigated, including the year in which the procedure was closed, the number of annexed TWRs, and the number of scanned pages for each case file in its entirety.

**Table 1.** Description of the case files investigated.

Abbreviation^2^	Canton	Year in which the procedure was closed	Number of annexed TWRs	Number of scanned pages of the whole case file
A1	A	2014	151	1218
A5	A	2011	240	4907
B6	B	2014	145	1046
B7	B	2018	198	2207
Total			734	9378

To trace how case file-makers refer to TWRs, we begin with an analysis of the basic question of the relationship between the total time of surveillance of conversations (for all phone numbers surveilled) per case file^3^ and the number of TWRs included in each case file. The content of the case files can be divided into the following types of documents: the *police report*, the written *reports of interrogations*, the *indictment*, the *judgment*, and other administrative documents, such as requests and approbations of authorizations for intercepting conversations, requests and approbations of authorizations for the transfer of the defendants between the prison and the courthouse, various bills, mail, and email exchanges between the different actors of the criminal procedure. For our purpose, the first four types are of interest because they are crucial for the respective case file makers (i.e. police, prosecutor, and judge).

Furthermore, these four types of documents were coded for other pieces of information necessary to answer our second research question, namely whether the representatives of the criminal justice authorities (e.g. the police officer, the prosecutor, or the judge) refer to the content of the intercepted conversations in the TWR by *citing* it in its entirety or partly, or by *summarizing* the content of those protocols and intermixing it with the police report or judgment. As our study reveals, *summarizing* is only one of the techniques used to integrate the TWRs into the case file. In fact, the way in which the content of the TWRs is referred to significantly impacts the visibility of the intercept interpreter as owners of that content. We use the term “owner,” which was specifically developed in the context of “interpreter-owned texts” (Mason and Ren, 2014: 124–125), to emphasize the fact that the intervention of interpreters can be made more or less visible (where visibility is seen as a continuum, see Angelelli, 2004: 76–77). More precisely, when citing a TWR, criminal justice authorities render the intercept interpreter’s ownership visible, and the content of the TWR is explicitly declared to be his or her product or output. The use of citations with quotation marks signals to the reader of the case file that the criminal justice authorities have not modified or revised the written TWR produced by the intercept interpreter. In contrast, when modifying the original TWR (using various methods, such as direct reported speech or summary), the intercept interpreter’s ownership is non-declared, as the criminal justice authorities do not use his or her product in its original form. By modifying the original TWR, criminal justice authorities erase the interpret interpreter’s agency in the criminal justice process.

Thus, depending on how the TWRs are referenced, the intercept interpreter’s ownership is either highlighted or concealed. To examine this question, we developed a coding scheme consisting of a fine-grained version with six categories and a coarse-grained version with two categories.

The fine-grained scheme consists of the following categories of label. The first label is *citation of seemingly word-for-word reports*, illustrated in example (1).^4^ This label was used to code cases in which certain parts are indicated to be word-for-word translations and transcriptions that use quotation marks when cited in police reports, indictments, reports of interrogations, and judgments. Still in example (1), the judge summarizes the conversation that took place at 20:56, in which s(he) includes the citation of a word-for-word report that the intercept interpreter produced. In this example, the judge also selects the content of the original TWR, which is signaled by an ellipsis. The length of a citation may vary between one word to one sentence or to the entire (brief) conversation, as shown below:

(1) *-at 20:56, P contacts S; a third party answers, and he tells him “please tell him to hurry up, some people continue to call me here [. . .] so I do not know what to do anymore”; the third party ensures him that S. will arrive shortly; [Judgment, A1]*

The second label corresponds to *seemingly word-for-word but enriched with focus techniques* and is illustrated in example (2). This label was used to code cases in which certain words or parts of sentences from a seemingly word-for-word report are marked with bold or italic fonts when they are included in a police report, indictment, report of interrogations, or judgment. In example (2), the text comes from a police report: the police wrote some of the words Y uttered (which the interpreter interpreted and wrote using a seemingly word-by-word method in the TWR) in bold to indicate that they are particularly important for the point the police report makes.

The same example also illustrates the third label: the *selection of content by using ellipses (*“. . .”*)*. This label is used to code cases when criminal justice agents select part of a report on transcribed conversations in the police report, the indictment, the report of interrogations, or the judgment while skipping content:

(2) *Y : Did you finish with her?*
 *X*: ***Yes, she has left to Zurich. She told me that she had bought 50 fingers in Lisbon and that she has returned with them, that she will give them to one of her clients.***
 *Y: Tell me how many did she give you?*
 *X: The small ones are 24, the big ones 5, I will take them apart tomorrow.*
 *B*: ***It is a heavy deal, very strong*** *but I do not think that we can talk of everything right now*
 *. . .*
 *Y*: ***In total there are 412***. *[Police Report, B7]*

The fourth label is *summary* and is illustrated in example (3). This label was used to code cases in which certain parts of a conversation seemingly translated and transcribed word for word are summarized in the police reports, indictments, reports of interrogations, or judgments. The fifth label, illustrated in example (4), concerns *direct reported speech*. This label was used to code cases in which an initially seemingly word-for-word report is reformulated and conveyed as direct (reported) speech:

(3) *In a subsequent telephone conversation, S explains to M that the mule is a train worker and that he is coming to L. He asks him if he can’t find a car to pick him up. When the latter is reluctant, S points out that they are cowards, and that the transporter working on the trains makes deliveries every week, to which M replies that he will try to find someone to take him to L. S then asked him why he was “stuck like that.”* [Judgment, A5]
(4) *-at 11:37, P contacts C to ask him “you can have how much money I want to know”; the other one answers “I will give you everything”;* [Judgment, A5]

The sixth, and last, label is *reference to quantities* and is exemplified in (5) below. This label was used for cases in which the only part of the conversation referred to in a police report, indictment, report of interrogations, or judgment is the quantity of drugs or of money. In other words, the events described in the conversation are omitted, and the only relevant information to be reported refers to the quantity.

*(5) Telephone conversations between S and B confirm this delivery of drugs, with S specifically mentioning 380 grams in a telephone conversation with M on xx 20xx at 22:45.* [Judgment, A5]

In sum, this fine-grained coding scheme was used to examine ways of documenting references to intercept interpreters’ output, the TWR. Data from the four main sections of a case file—indictments, police reports, reports of interrogations and judgments—was coded according to this fine-grained coding scheme. This represents a total of 1171 data points.

In the coarse-grained version of the coding scheme, we narrowed the research focus to a binary distinction: the presence or absence of visible ownership. In other words, our coding scheme distinguishes between *declared* ownership and *non-declared* ownership of the initial TWR about the intercepted conversation. More precisely, when the interpreters’ output was documented only by means of verbatim citations of the original TWRs, the ownership of the content referred to is declared, and thus the interpreters’ co-authorship is rendered visible. In contrast, when the interpreters’ output was documented by means of the categories 2 to 6 (*summary, direct (reported) speech, selection of content, reference to quantities*, and *word-for-word enriched with focus techniques*), as well as citations of the original TWRs that co-occur with any of these strategies, their co-authorship is non-declared and made invisible. Furthermore, if a verbatim citation of an original TWR was accompanied by any category from 2 to 6 from the fine-grained scheme (such as in example 2, where the *selection of content by using ellipses* appears with the word-for-word text), the data point was coded as non-declared ownership.

## Findings

We first asked the question about the relationship between the total time during which conversations were surveilled (for all phone numbers) per case file and the number of protocols (TWRs) included in each case file. The results are shown in Table 2: the number of TWRs included in the case file is likely much smaller than the total number of intercepted conversations during the time of surveillance in each case file.

**Table 2.** Total time of surveillance measures versus number of TWR.

	A1	A5	B6	B7
Total time of surveillance	5 weeks and 5 days	150 weeks and 2 days	20 weeks	23 weeks
Number of TWRs in the case file	151	240	145	198

This is particularly striking in the A5 case file, in which only 240 TWRs were included, while the monitored suspect’s conversations were intercepted over 150 weeks and 2 days. In other words, the number of intercepted conversations is much more important than the number of reports on translated conversations included in the case file by means of TWRs. Furthermore, it is revealing that audio files of the intercepted conversations are transmitted on CD-ROMs, but the documents of the case files almost never contain references to the audio-recordings or to the annexed TWRs. Sometimes, there is an explicit indication that more details are available for readers, but there is no reference to a specific page. Most often, however, the relevant excerpt of the TWR is referred to in the police report, the report of interrogations, or to the judgment itself—a finding that highlights the importance of examining how it is referenced.

**Figure 1.**
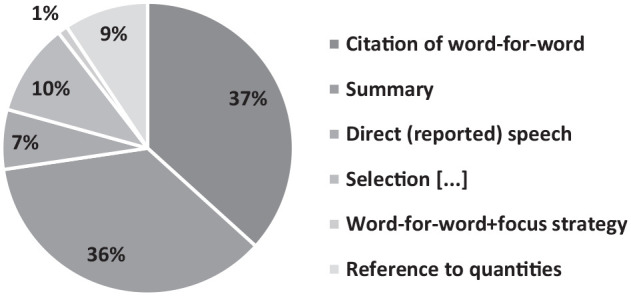
Methods of referring to translated and transcribed content of intercepted conversations.

Furthermore, Figure 1 reveals the frequency of the fine-grained labels (citation of seemingly word-for-word, summary, direct [reported] speech, selection of content, reference to quantities, and word-for-word enriched with focus techniques) in the four case files examined (in all the documents of the case files, with the exception of the annexed TWRs).

Our data suggests that the most frequent ways of referring to the content of TWRs are the word-for-word citations with quotation marks (631 data points, 37%), followed by summary (618 data points, 36%); reference only to quantities (of money or drugs; 161 data points, 9%); direct reported speech (114 data points, 7%); and word-for-word enriched with focus techniques (19 data points, 1%). In 10% of the cases, the content of the TWR is referred to only partially (176 data points), that is, only some parts of the conversation were selected and referenced. The techniques used to refer to the content of a TWR in the case file may also co-occur. For example, citations of word-for-word content may co-occur with a summary or with an ellipsis indicating the selection of the most relevant content or with focus techniques, as in example (2).

Interestingly, the frequency of these strategies depends on the section of the case file. Table 3 shows how these methods are used in each section of the case files analyzed.

**Table 3.** Methods of referring to translated and transcribed content of intercepted conversations.

	Citation of word-for-word	Summary	Direct (reported) speech	Word-for-word and focus strategy	Reference only to quantities	Selection [. . .]	Total
Indictments	0	8	0	0	5	0	13
	0%	61.5%	0%	0%	38.5%	0%	100%
Judgments	257	239	114	0	127	82	819
	31.4%	29.2%	13.9%	0%	15.5%	10%	100%
Police reports	180	306	0	16	28	87	617
	29.2%	49.6%	0%	2.6%	4.5%	14.1%	100%
Reports of interrogations	194	65	0	3	1	7	270
	71.9%	24.1%	0%	1.1%	0.4%	2.6%	100%

In particular, we find that the way the case files refer to TWRs changes over the course of the procedure: at the beginning, police reports are produced, and in police reports, TWRs are most frequently referred to by means of summaries (49.6%) and word-for-word citations (29.2%), while focus strategies applied to word-for-word translated and transcribed content (2.6%) and reference to quantities (4.5%) are used less often. In 14.1% of the cases, we find that the content referred to is partial. In interrogation records written by police officers or prosecutors, TWRs are most frequently referred to by means of citations (71.9%) and summaries (24.1%). At the end of the investigation phase, in indictments—that is, the document prosecutors write and that contains the charge or charges the defendant faces at trial—the most frequent ways of referring to TWRs are summarizing (61.5%) and referring only to quantities (of money or drugs; 38.5%). Finally, in judgments, TWRs are referred to with word-for-word citations (31.4%), summaries (29.2%), direct speech (13.9%), and by references to quantities (15.5%). The content referred to is signaled as being only partial with an ellipsis in 10% of the data points. One notable example is that of judgments. In the case files investigated, judges systematically draw on the content of TWRs to make a judgment. They may cite seemingly word-for-word content found in the TWR; summarize it; reformulate it as direct (reported) speech or crystallize it to keep only its essence, that is, the main events and/or the quantities of drugs or money involved; and, finally, they may select only the passages most relevant for their judgment.

By using these strategies, criminal justice agents frequently disguise the text ownership of intercept interpreters, thus rendering their co-authorship quality invisible. To illustrate this discrepancy between the content of the intercepted conversation contained in the original TWR and the way in which criminal authorities refer to it, we provide examples (6)−(11). Examples (6) and (7) have the same content of an intercepted conversation but presented in different forms: in (6), the content comes from the police report, whereas in (7), the content comes from the original TWR.

(6) *S calls an unknown African person. The unknown person has recognized that he received 13′000 CHF, then 4′200 CHF, whereas, in total, the money was 16,500 euros, so due to the exchange, money misses.* [Police Report, A5]
(7) *Conversation of 12.09.2011 at 22:04.*
 *From X to* Unknown *African person*
 – *hello*
 – *yes here B; how is it?*
 – *hello*
 – *yes*
 – *yes*
 – *how is it? (anything new)*
 – *ah?*
 – *anything new?*
 – *ah, peace only, do you have peace there also?*
 – *ah a little, little (step by step)*
 – *ok*
 – *yes*
 – *do you hear me because it is noisy; where are you? I do not hear you well*
 – *oh no I am opening a door for someone huh*
 – *Ah oh*
 – *hmm*
 – *ah yes it is H who told me to call about the thing there huh*
 – *ah yes the papers are not complete huh*
 – *ah, ha*
 – *yes you know there was 16 and 500 in our money here*
 – *yes hmm*
 – *yes so the other one 13 that they gave there*
 – *hmm*
 – *oh yes I thought that it was 13 of the money that we have here the 13*
 – *hmm yes*
 – *well me the other one since they gave him me it was yesterday that I received the papers in my hand my friend there it is in Zurich the one that has the shop. It is yesterday that he came here. He brought them. Huh I thought that it was our papers that he brought. Huh, he owes me some papers. Then we started to discuss, we discussed, and it is then that he told me: no, it is not our papers, that they have their papers your papers these.*
 – *hmm*
 – *ok, then, I told: no, these are not our paper because H even asked me last days how much, how much it was left. it is 3500 that was left.*
 – *hmm*
 – *because I thought that it was our papers here, that they gave 13 but in fact it is your papers there that they gave 13 and then 4,200 more*
 – *hmm*
 – *so, this does not sum up to 16,500 et this is what it should be. Then what is this?*
 – *ah, you know before he received, he went to ask for the rate, huh, was is the rate because you know the young did not have documents to make the exchange, huh, so he said that in Zurich he will do the exchange, huh.* [TWR, Annexes, A5]

This example shows how criminal authorities do not provide the original, word-for-word, TWR but summarize it by reporting only the main events and the quantities of money involved.

Another strategy criminal authorities use to refer to the content of a TWR is to provide the word-for-word TWR enriched with focus techniques. This is illustrated in examples (8) and (9); the former is from a police report, while the latter is from the TWR.

(8) *The below conversations make us believe that C had to obtain coke from U with whom he was friend.*

*10.07.2017 at 21:20, U calls C:*

 – *Yes?*
 – *Are you gone? I think that by Friday I will want to leave from here, as soon as I will be ready on the other side because the sending of the money from here will be difficult, furthermore it is difficult to find a person to send the money, I have to arrive to Portugal to make it.*
 – ***I think that by Friday we will have something then we will see each other. For the moment, I am waiting to collect (interpreter’s note: he speaks of money).***
 – ***This is why I tell you that Friday, like this I can bring the utmost.*** *Can you find someone to send, it is difficult and I do not want to wait until there is nothing left (interpreter’s note: he speaks of drugs) to start searching, it will delay me. I would not like to come back, in this way* ***I can send a person to see you***, *and I will prepare me for my travel. I will see by Friday how I will have sorted out on my side, ok.*
 – *Keep me informed.* [Police report, B7]
(9) *U calls C*
 – *Yes?*
 – *Are you gone? I think that by Friday I will want to leave from here, as soon as I will be ready on the other side because the sending of the money from here will be difficult, furthermore it is difficult to find a person to send the money, I have to arrive to Portugal to make it.*
 – *I think that by Friday we will have something then we will see each other. For the moment, I am waiting to collect (interpreter’s note: he speaks of money).*
 – *This is why I tell you that Friday, like this I can bring the utmost. Can you find someone to send, it is difficult and I do not want to wait until there is nothing left (interpreter’s note: he speaks of drugs) to start searching, it will delay me. I would not like to come back, in this way I can send a person to see you, and I will prepare me for my travel. I will see by Friday how I will have sorted out on my side, ok.*
 – *Keep me informed.* [TWR, Annex, B7]

Examples (8) and (9) show that criminal authorities cite the content of the original word-for-word TWR, in which they add focus techniques, such as bold-face type. Interestingly, in this example, we can also see two cases in which the intercept interpreter specifies further, first that the conversation is about money, and second that it concerns drugs. Nevertheless, criminal authorities do not explicitly mention this in their report, nor do they mention the fact the citation contains the intercept interpreter’s output (i.e. the TWR).

Another example comes from an indictment that contains no citations from the original TWRs. Furthermore, the entire argumentation is built on several intercepted conversations but does not refer to those conversations. Example (10) is an excerpt from the indictment against S (the B7 case file), and example (11) comes from the indictment against O (the A1 case file).

(10) *S has intentionally, in agreement with other persons among which H and B, participated in a large-scale drug trafficking operation by giving numerous and precise instructions over the telephone to his various accomplices, at all hours of the day and night, in order to actively organize the financing, importation, delivery, and distribution from his cell in B Prison.* [Indictment, B7]
(11) *O has intentionally participated, in A-city (name of the city replaced by the authors for anonymization reasons), in an important drug trafficking: 1. Between 9 and 15 January 2014, by organizing the import in Switzerland, then the transport in this country, of minimum a quantity of 560 grams of coke, intended for selling. For this, he has first collected money from his companions, that is S, U, O, A, N, L, and T in A-city and in the canton Vaud, then he travelled to Amsterdam, probably on January 9 to resolve organizational and financial matters, before coming back to A-city, by place, on January 11, 2014.* [Indictment, A1]

**Figure 2.**
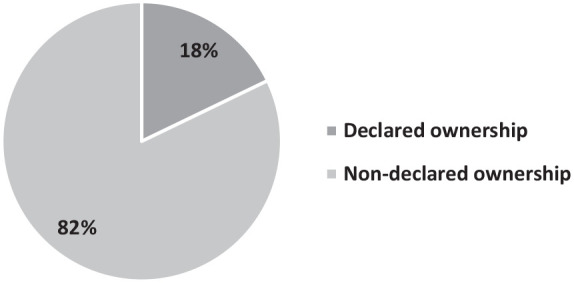
Frequency of declared versus non-declared ownership of the content referred to.

As explained above, the coarse-grained coding scheme distinguished between cases in which interpreters’ co-authorship was declared or, on the contrary, was not declared. This coarse-grained analysis revealed that, among all the case files examined, the source of the content is declared in only 18% of cases (209 occurrences of citations of word-for-word reports). In the majority of cases, the intercept interpreter’s involvement is not declared: in the remaining 82%, that is 962 occurrences, linguistic means other than verbatim citations of non-modified TWRs were used, as illustrated in Figure 2. The distribution of observed values regarding a declared versus non-declared source is statistically significant rather than being due to chance (χ^2^ = 481.64, *df* = 1, *p* < 0.0001).

Hence, all these examples, which do not represent isolated cases as confirmed by the results of our quantitative analysis, disclose how criminal authorities render invisible the intercept interpreter’s role as co-author. It is only after having already gradually built the master narrative that criminal justice agents, occasionally, test the robustness of the story by confronting defendants with extracts of the TWR. At this point only, the authorship of the intercept interpreters is unveiled in the related documents, that is the written reports of interrogations. Once the narrative is constructed and presented in court, judges are more willing to reveal intercept interpreters’ co-authorship by directly using the content of TWR, though to a limited extent, mainly by indicating the main events and figures (e.g. quantities of drugs or money). Indeed, in strong contrast to indictments, for instance, in judgments, references to the intercepted conversations and their content are very frequent. More precisely, judges build and explain their judgment by making explicit reference to the fact that the evidence comes from the intercepted conversations, as in example (12). Judges refer to their content by selecting and citing only very crucial parts of these conversations, by summarizing other parts, and by using direct speech, as shown in example (13):

(12) *The following elements come out of the intercepted conversations.* (Judgment, A1)
(13) *Facts from 16 February 2014*
 *At 11:17, A calls O to speak about money; the latter tells his “I will call C to see if he has some money you understand” and O concludes that “even if I am alone, I will do it step by step”;*
 *at 11:37, O contacts C to ask him “how much money can you get out, I want to know”; this one answers, “I will give you everything”;*
 *at 14:35, O calls someone named B, telling him, “I need help now, I do not know how to make [. . .] for the money. . .only as usual, please”;*
 *between 17:20 and 18:30, O sends A to B so that the latter give him money;*
 *at 19:08 A calls O, who is at the railway station.* (Judgment, A1)

To better compare this extract from the judgment with the original TWRs referenced, below, in example (14), we provide the TWR of the conversation that took place at 11:17.

(14) *A calls O*
 – *Hello O*
 – *Hello*
 – *Yes, how are you?*
 – *Good, and you?*
 – *Good, did you wake up?*
 – *Yes*
 – *Is everything ok, I want to know (not very clear)*
 – *What do you say?*
 – *I say that I do not know how.. have you money with you?*
 – *No*
 – *Did that guy give you this money?*
 – *He called me the other day and told me that he will be evicted from the center, you understand?*
 – *Yes*
 – *So, he does not have money, the little money that he had he used to go to Italy . . . you know. . .he will call me tomorrow morning*
 – *Ok, I will call C to see if he has some money you understand*
 – *Yes*
 – *Try to call O and speak with him, you understand what I want to say*
 – *I will call him to speak with him but is it with S*
 – *What?*
 – *S*
 – *Yes.*
 – *You ask me to do what?*
 – *I tell you that it is for S*
 – *Yes it is this that I speak*
 – *It is better S*
 – *Good*
 – *Even if I am alone, I will do it step by step*
 – *It is true*
 – *Indeed*
 – *It is for people of..*
 – *Indeed, you know S, it is the one without problems. We will do it step by step*
 – *Ok*
 – *Ok, then you call him*
 – *Good*
 – *Ok*
 – *See you soon* [TWR, Annex, A1]

Examples (13) and (14) are typical instances found in our data set (as shown by the quantitative analysis) and demonstrate how judges reveal the intercept interpreter’s co-authorship by citing seemingly word-for-word content found in the TWR. Thus, the interpreters’ co-authorship is recognized and declared. At the same time, judges also summarize the original content of the TWRs, reformulate it as direct (reported) speech, crystallize it to preserve only its essence, and then select only those passages that are most relevant for their judgment. In this way, judges, similar to the other two bodies of criminal authorities (the police and prosecutors), render invisible the interpreters’ co-authorship of the narrative built in the case file.

## Concluding discussion

This paper aims to increase our understanding of the role that intercept interpreters can play when collecting evidence. This shapes the way in which a case narrative develops. The selection, extraction, and editing of material from large collections of surveillance content clearly involves a form of co-production between investigators and intercept interpreters. This co-production of the narrative implies co-authorship. Our data and the narrative criminological approach endorsed in this study provide an original contribution to the state of research about critically reconstructing narratives on the basis of case files. Our results bring into light the role of intercept interpreters: active actors who are co-authors of the master narrative deployed in a criminal procedure. By examining how authorities refer to the intercept interpreters’ TWRs in four important document types of a case file (i.e. the police report, the interrogation report, the indictment, and the judgment), our analysis reveals a clear shift between the initial construction of the narrative by the investigating police and prosecutorial services and the later stages of the case, when the narrative is constructed and presented in court. In what follows, we discuss this shift.

During the initial construction of the narrative by the police and the prosecutorial services, the co-authorship of intercept interpreters is highly important because the case is built on the basis of the intercepted conversations and their corresponding TWRs. However, the ownership of intercept interpreters is specifically rendered invisible in the documents of these authorities (police reports and indictments). In fact, we found that, overall, the strategy criminal justice agents used to refer to the intercept interpreters’ output, that is the TWR, is most often by means of summaries, direct (reported) speech, word-for-word enriched with focus strategies, and reference only to quantities (of drugs or money).

From the point of view of the socio-legal insight regarding performative writing (Vismann, 2008: 56), the fact that criminal justice authorities render the interpreter’s co-authorship invisible is disturbing: when creating TWRs, intercept interpreters exercise performative writing, which is a fact-producing act. Though their performative writing, intercept interpreters establish “reality.” TWRs become a “factual” account of what happened during intercepted conversations. Hence, despite the importance of the act of co-authoring the initial construction of the narrative, this involvement is not as openly disclosed as it could be. We explain this elision of the intercept interpreters’ authorial contributions to the master narrative as stemming from a lack of professional self-perception among intercept interpreters (Capus and Havelka, 2022). In addition, the legal setting in criminal procedures empowers the police and the prosecutor to investigate and construct the story of the case in line with the legal script. Intercept interpreters have neither a criminalistic nor a legal background; they exclusively have the linguistic competence to understand the intercepted communications. Indeed, the conventions of legal narratives told in the specific setting of a criminal procedure govern not only the narratives that are appropriate or permitted, but also how these stories are told, including who may tell them, the amounts and types of information included and their relevance (Bandes, 1996: 384). With respect to intercept interpreters, criminal justice maintains the fiction of the mere transfer of contributions, without any autonomous decisions regarding the selection of the content of the TWR (Capus and Griebel, 2021). Through this, they fail to clearly identify the intercept interpreters’ role as co-authors. These are impactful silences (see Presser and Sandberg, 2019 about silenced stories with regard to antisociality) in the dominant documented narrative about investigating foreign language-speaking suspects. By blurring the agency of intercept interpreters, criminal justice agents whitewash actual unsolved questions of accountability between police and intercept interpreters regarding the selection and the editing of intercepted conversations. Telling the story in this way maintains the current state of affairs and perpetuates the fictional ideas that police agents and prosecutors lead, define and control the initial plotting, and that they are the only reliable curators of the evidence gathered (see Offit, 2019: 54 regarding this self-representation in prosecutors’ oral contributions).

Our data shows that case files are not only an important resource for discovering the storytelling about the investigated crime, but also they contain the narrative about investigating, prosecuting and judging with the help of intercept interpreters. The way in which storytelling is performed most often renders invisible the interpreters’ co-authorship of the narrative built in the case file. This finding is troubling since research in monolingual interception has already shown a strong police bias embedded in these activities (Nunn, 2010: 32; Bucholtz, 2009; Shuy, 2005; Tiersma and Solan, 2005: 192). Our data supports these previous findings and complete them by showing that, as soon as the activities of intercept interpreters, such as selecting and editing the content of the intercepted conversations, are rendered invisible, they become a bias embedded in the investigation.

This proposal is concurrent with other previous observations, according to which elements of counter-narratives are not likely to be integrated into the case file containing the master narrative. According to Bucholtz (2009: 507), a “bias in legal transcription in favor of those who hold the greatest institutional power—judges, lawyers, and police officers”—is a consistent pattern when it comes to transforming speech into text in legal settings. Hence, to develop a counter-narrative, defendants and defense lawyers in criminal proceedings with intercept interpreters have a particularly difficult task: challenging the master narrative not only requires understanding the original conversation and showing that TWRs have been truncated or summarized, but also that this may have been done in a biased way. For example, the interpreted conversations could have been misunderstood, in the sense that the discussion could have concerned the traffic of legal goods, such as cars or parts of cars, rather than of drugs. This could only be done by listening and translating the totality of intercepted conversations. However, resources to challenge or rebut the already established transcript evidence, and thereby the constructed narrative, often lack.

To conclude, in this paper, we have connected the area of case file research with that of narrative criminology. In doing so, we have established case files as important resources for storytelling and identified links with the research in narrative criminology that focuses on criminal justice agents (Offit, 2019; Petintseva, 2019; Ugelvik, 2016). Our approach enriches the state of the research with the findings that criminal justice agents, notably police and prosecutors, deploy a narrative in which they seldom recognize and declare the intercept interpreters’ co-authorship. Instead, they prefer to render their contributions invisible. This could be due to their self-conception of being the exclusive, neutral, and therefore reliable curators (Offit, 2017: 18) of the evidence.

## Notes

1. In Switzerland lawful interception has to be approved by a court (Art. 272 CrimPC; Swiss Criminal Procedure Code, fedlex.admin.ch/eli/cc/2010/267/en, accessed 21 April 2021) and is mainly used in cases of drug trafficking (Art. 19 Narcotics Act; Federal Act on Narcotics and Psychotropic Substances, fedlex.admin.ch/eli/cc/1952/241_241_245/en, accessed 3 August 2021), organized crime (Art. 260^ter^ CrimC; Swiss Criminal Code, fedlex.admin.ch/eli/cc/54/757_781_799/en, accessed 27 July 2021), human trafficking (Art. 182 CrimC), or terrorism (Art. 260^quinquies^, 260^sexies^ CrimC). Within Swiss criminal proceedings, communications surveillance is part of the investigation conducted by the police under the lead of the prosecutor during the preliminary proceeding phase (Art. 308 ff. CrimPC, Art. 269 ff. CrimPC).

2. The abbreviation used in this article refers to the number each case file has in the project’s research database.

3. We are thankful to our team colleague Elodie Bally for providing us with this information.

4. All examples are translated from French into English by the authors to illustrate the point made in this paper. In the case of the content of TWRs, the English translations may seem agrammatical and/or awkward. This has two causes. Firstly, they reflect the manner in which the presumed drug dealers communicate between them. Indeed, their speech is truncated or coded intentionally (to avoid being easily understood by third parties), and they understand themselves because they know about what they are talking about (shared background knowledge). Secondly, in some cases, the original French itself text is agrammatical because intercept interpreters are, in general, non-native speakers of French and their mastering of French is reduced. Thus, when translating into English, we chose to stay as closest as possible to the original French text so that we do not disrupt the empirical data.
